# A Genomic BSAseq Approach for the Characterization of QTLs Underlying Resistance to *Fusarium oxysporum* in Eggplant

**DOI:** 10.3390/cells11162548

**Published:** 2022-08-16

**Authors:** Maria Rosaria Tassone, Paolo Bagnaresi, Francesca Desiderio, Laura Bassolino, Lorenzo Barchi, Francesco Elia Florio, Francesco Sunseri, Tiziana Maria Sirangelo, Giuseppe Leonardo Rotino, Laura Toppino

**Affiliations:** 1 Council for Agricultural Research and Economics, Genomics and Bioinformatics Research Center, 26836 Montanaso Lombardo, Italy; 2 Department of Agricultural Science, University Mediterranea of Reggio Calabria, 89124 Reggio Calabria, Italy; 3 Council for Agricultural Research and Economics, Genomics and Bioinformatics Research Center, 29017 Fiorenzuola d’Arda, Italy; 4 Council for Agricultural Research and Economics, Cereal and Industrial Crops Research Center, 40128 Bologna, Italy; 5 DISAFA, Plant Genetics and Breeding, University of Turin, 10095 Grugliasco, Italy

**Keywords:** *Solanum melongena*, soil-borne fungal pathogens, recombinant inbred lines (RILs), molecular mapping, biotic stress, resistance genes

## Abstract

Eggplant (*Solanum melongena* L.), similar to many other crops, suffers from soil-borne diseases, including *Fusarium oxysporum* f. sp. *melongenae* (*Fom*), causing wilting and heavy yield loss. To date, the genetic factors underlying plant responses to *Fom* are not well known. We previously developed a Recombinant Inbred Lines (RILs) population using as a female parent the fully resistant line ‘305E40’ and as a male parent the partially resistant line ‘67/3’. The fully resistant trait to *Fom* was introgressed from the allied species *S. aethiopicum*. In this work, the RIL population was assessed for the responses to *Fom* and by using a genomic mapping approach, two major QTLs on chromosomes CH02 and CH11 were identified, associated with the full and partial resistance trait to *Fom*, respectively. A targeted BSAseq procedure in which Illumina reads bulks of RILs grouped according to their resistance score was aligned to the appropriate reference genomes highlighted differentially enriched regions between resistant/susceptible progeny in the genomic regions underlying both QTLs. The characterization of such regions allowed us to identify the most reliable candidate genes for the two resistance traits. With the aim of revealing exclusive species-specific contigs and scaffolds inherited from the allied species and thus associated with the full resistance trait, a draft *de-novo* assembly of available Illumina sequences of the ‘305E40’ parent was developed to better resolve the non-recombining genomic region on its CH02 carrying the introgressed *Fom* resistance locus from *S. aethiopicum*.

## 1. Introduction

The large family of the Solanaceae comprises over 3000 plant species adapted to a wide range of geographic conditions, including the cultivated eggplant (*Solanum melongena* L.), tomato (*S. lycopersicum* L.), potato (*S. tuberosum* L.), pepper (*Capsicum annuum* L.), and tobacco (*Nicotiana tabacum* L.). In contrast to many species belonging to this family, eggplant originated from the Old World. Contrasting conclusions about the origin of domesticated eggplant have been reported by several authors, especially for single vs. double independent origins [1,2,3,4,5]. The common eggplant, also known as “brinjal” or “aubergine,” represents the third most important crop of the Solanaceae family, after potato and tomato. It is cultivated worldwide, with a global production of 56 Mt in 2020 [6]. Asia represents the main area of cultivation (93% of both the world production and harvested area, with China and India as the main producers), followed by Africa and the subtropical regions. In the Mediterranean basin, the crop is mainly cultivated in Egypt, Turkey and Italy [6]. Two allied species of common eggplant, scarlet (*S. aethiopicum* L.) and gboma (*S. macrocarpon* L.) eggplants, are native and commonly cultivated in Africa, while locally cultivated in other countries, including Italy.

Eggplants are susceptible to many diseases and particularly to some soil-borne fungal wilts caused by *Fusarium oxysporum* f. sp. *melongenae* (*Fom*), which is responsible for one of the most devastating vascular wilt diseases in eggplant [7,8,9,10]. The fungus penetrates through the roots and proliferates in vascular tissue. Wilting progresses from the lower to the upper leaves, causing yellowing to necrosis, followed by the collapse of the plant, especially when young plantlets are attacked. *Fom* has been identified in both open field and greenhouse cultivation in several countries, affecting eggplant production and causing heavy yield losses [7,10,11]. Despite the anthropogenic selection causing a drastic reduction of the genetic variation in the cultivated germplasm [12,13], partial resistances to most pathogens were found within the eggplant gene pool, with the degree of resistance often scarce for effective employment in breeding programs [12]. Nevertheless, some promising resistance traits to *Fusarium* wilt have been identified in *S. melongena* [14,15,16,17,18] and successfully transferred into breeding lines with the development of associated molecular markers [19,20]. On the other hand, it is well known that *S. melongena* progenitors, allied and wild relatives are important reservoirs of potential genetic variability for many agronomic and qualitative traits, as well as a source of valuable resistance to diseases and pests [21,22,23,24]. For this reason, conventional (sexual crosses) and unconventional (protoplast fusion, embryo rescue) strategies to introgress traits of interest into the genetic background of cultivated eggplant [13,25], including full resistance to *Fusarium oxysporum* [26,27,28,29,30], have been employed.

Some studies aimed to characterize the eggplant defense responses and signaling pathways activated upon *Fom* infection, as well as identifying loci, QTLs and genes involved in the resistance to fungal wilts, have been carried out [18]. From a cross with an Asian accession [16], eggplant breeding lines resistant to *Fom* were developed and characterized by different molecular markers associated with the resistance trait [19], which therefore proved to be useful in assisting breeding through MAS [31,32]. Toppino et al. (30) demonstrated that the *Fom* resistance trait introgressed into eggplant from protoplast somatic hybridization with *S. aethiopicum* and *S. integrifolium* is controlled by a single dominant locus (named *Rfo-sa1*) and developed codominant molecular markers associated with the resistant and/or the susceptible phenotype through a Bulked Segregant Analysis (BSA) [33].

The *Rfo-sa1* locus was afterwards localized on the eggplant chromosome CH02, using a RAD-tag-derived markers map [34] based on the intra-specific segregant F2 population from the cross ‘305E40’x’67/3’, in which the male line, ‘67/3’ was recently employed to develop a high-quality genome sequence [35], while the female parent, ‘305E40’, is an introgressed, double-haploid line developed by somatic hybridization with *S. aethiopicum* [26,30] and is fully resistant to *Fom*. The genotypic characterization of ‘305E40’ line revealed a haplotype identical to that from *Solanum aethiopicum* on the short arm of chromosome CH02, which included the locus *Rfo-Sa1* [36]. This “alien portion” was probably inherited from the allied species during the production of the somatic hybrid through protoplast fusion. Two major QTLs for the resistance trait to *Fom* were identified in the same F_2_ population: the first QTL on chromosome CH02, *FomE02*, derived from the resistant parent ‘305E40’ lying in the genomic region of the *Rfo-Sa1* locus inherited from *S. aethiopicum*, and a second one, *FomE11.1*, on chromosome CH11, inherited from the male parent ‘67/3’ which carries a source of partial resistance to *Fom* never spotted before [18]. Two *Fusarium* semi-dominant inherited resistance loci [37,38] were also mapped on chromosomes CH02 and CH04 in a segregant population developed from Asian *Fom*-resistant lines (with the QTL on CH02 orthologous to the *Rfo-sa1* locus), and a set of orthologous candidate genes was proposed by exploiting the syntenic relationships with tomato [20]. Moreover, candidate genes involved in early defense responses or signaling pathways activated upon infection were identified in the *Fom*-resistant advanced breeding line ‘305E40’ [39].

Recently, a F_6_–F_7_ RIL population was developed from the same cross ‘305E40’x’67/3’, whose 5X Illumina sequencing data, together with an Illumina 35X sequencing of ‘305E40’ line were exploited to anchor the scaffolds of the sequenced line ‘67/3’ to the 12 chromosomes to obtain the first anchored eggplant genome sequence [30]. A high-density map based on genotype-by-sequencing (GBS) was more recently developed on the same population. It has been successfully used to confirm and better define several QTLs already mapped on the F2 population from the same cross and to identify new regions associated with plant anthocyanin content and seed quality traits as well as fruit metabolomic content and anthocyanin peel coloration [40,41,42,43]. A more continuous Hi-C-based assembly of the line ‘67/3’, together with the first pangenome of eggplant obtained by resequencing 23 additional accessions of *S. melongena*, was also even more recently released [44].

In the present work, this high-density GBS-based map was exploited with the aim to detect new QTLs (or to better define the already mapped ones), as well as to identify the most reliable candidate genes presiding the full and partial resistance traits to *Fom* inherited from ‘305E40’ and ‘67/3’. 

## 2. Materials and Methods

### 2.1. Plant Material

A population of 168 F_6–7_ Recombinant Inbred Lines (RIL), recently characterized by GBS [18], was employed in this work. The RIL population was developed from the cross between the two eggplant breeding lines ‘305E40’ and ‘67/3’, contrasting for a wide number of key agronomic and metabolic traits [18,34,36,45]. The line ‘305E40’ (female parent) is a double haploid derived from an interspecific somatic hybrid *Solanum aethiopicum* gr. *gilo* (+) *S. melongena* cv. Dourga [26], which was repeatedly backcrossed with the recurrent lines ‘DR2’ and ‘Tal1/1’, before selfing and anther culture. This breeding line carries the locus *Rfo-sa1* introgressed from *S. aethiopicum*, which confers complete resistance to the soil-borne fungus *Fom* [18,30]. Otherwise, the line ‘67/3’ is a F_8_ selection from the intra-specific cross between *cvs* ‘Purpura’ and ‘CIN2′, that showed a partial resistance trait to *Fom* [18].

### 2.2. Eggplant/Fusarium Oxysporum f. sp. Melongenae (Fom) Resistance Assessment

To assess both the full and partial *Fom* resistance traits, the RILs, together with parental lines, their F_1_ hybrid and the full susceptible (line ‘Tal1/1’) and resistant (*S. aethiopicum*) control materials were sown in plastic trays filled with pasteurized peat and grown in greenhouse at the Research Centre for Genomics and Bioinformatics, Montanaso Lombardo (45°20′ N, 9°26′ E). For each progeny/accession, a 104-hole tray was used. A total of 8135 plantlets were employed to assess resistance to *Fom*. Each progeny and control line was assessed for the presence/absence of the *Rfo-Sa1* locus, strictly linked to the full resistance trait inherited from *S. aethiopicum* in the resistant line ‘305E40’. To this end, two plants of each progeny were assessed through genotyping with molecular markers linked to the locus, as described by Toppino et al. [30] (data not shown). 

The inoculation was conducted according to the dip-root method reported by Cappelli et al. [46]. Plantlets, at the 2–3 true leaf stage, were gently removed from the tray and their roots washed under running tap water and then immersed for 15 minutes in a conidial suspension of *Fusarium oxysporum* f. sp. *melongenae* at a concentration of 1.5 × 10^6^ conidia/mL. All plantlets (min 22, max 74 plants) for each progeny were divided into two blocks and then inoculated with the *Fom* conidial suspension. After dipping, the two blocks of plants were transplanted into 54-hole trays and randomized in two different greenhouses until symptom evaluation. For each line and progeny, 9 plants were mock inoculated with water and kept in a greenhouse as a negative control. 

**Figure 1:**
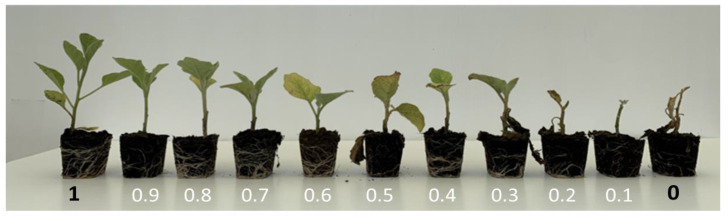
Visual representation of the degree of symptoms assigned to each single plant, ranging from 1 to 0.

Evaluation of symptoms was assessed on each plant 30 days after inoculation (DAI), according to a scale (compared with mock inoculated controls, from Barbierato et al. [39]) ranging from 1 to 0, where 1 corresponds to “fully resistant plant with complete absence of symptoms”, 0 to “dead plant”, and with the intermediate values as follows: 0.9 = some yellowing spots in basal leaves, absence of symptoms in intermediate and upper ones; 0.8 = extended yellowing in basal leaves; 0.7 = extended yellowing in basal leaves and some yellowing spots in intermediate ones; 0.6 = extended yellowing in both basal and intermediate leaves; 0.5 = some necrosis spots in basal leaves, extended yellowing in basal and intermediate ones and some spots of yellowing in upper ones; 0.4 = partial necrosis in basal leaves, extended yellowing in intermediate and upper ones; 0.3 = necrosis in basal leaves and some necrosis spots in intermediate ones; 0.2 = necrosis in basal and intermediate leaves, falling of basal ones; 0.1 = complete necrosis in all the leaves, falling of basal and intermediate ones (Figure 1).

For each block, the resistance ratio (%) was calculated as:

$$R=\frac{\sum \;\left(\mathrm{plant}*\mathrm{score assigned}\right)}{\mathrm{total}\;\mathrm{no}.\;\mathrm{of}\;\mathrm{inoculated}\;\mathrm{plants}}\times 100.$$

### 2.3. Statistical Analyses and QTL Detection

Analysis of variance (ANOVA) of the resistance trait was performed to test the significance of differences between RILs and replications using JMP v. 7 software [47] (SAS Institute, Milano, Italy). Broad-sense heritability values were given by σ2G/([σ2G + σ2E]/n), where “σ2G” represented the genetic variance, “σ2E” the residual variance and “n” the number of replicates.

Normality, kurtosis and skewness were assessed with the Shapiro–Wilks test (α = 0.05). Segregation was considered transgressive when at least one RIL recorded a trait value higher or lower by at least two standard deviations than the higher- or lower-scoring parental line.

QTL mapping was conducted using a recently published high-density genetic map [18] with the R/qtl package of the R statistical computing software [48]. For each trait, an initial QTL scan was performed using simple interval mapping with a 1 cM step [49], and the position of the highest LOD was recorded. A genome-wide significance level of 5% was calculated after 1000 permutations [50], and the LOD threshold was used to identify a QTL. The QTL location and effect were then determined using the multiple imputation method by executing the “sim.geno” command, followed by the “fitqtl” command [51]. To search for additional QTLs, the “addqtl” command was used. If a second QTL was detected, the “fitqtl” was used to test a model containing both QTLs and then the interaction effect. If both QTLs remained significant, the “refineqtl” command was used to re-estimate the QTLs’ positions based on the full model, including both loci. QTL interactions were studied, and the significant locus combinations were reported based on the F-measure. The additive effects of QTLs were estimated as half the difference between the phenotypic values of the respective homozygotes. The confidence interval (CI) of each QTL was determined as proposed by Darvasi and Soller [52].

### 2.4. Composition of the Bulks of 5X Illumina Sequences

Three bulks (RR, resistant; SS, susceptible; PR, partially resistant) of available 5X Illumina sequences for each RIL (from Barchi et al. [35] submitted to the NCBI Sequence Read Archive under accession number SRP078398) were clustered according to their disease symptoms score:

1. BULK RR includes 28 fully resistant RILs with a calculated disease ratio of 100% and harboring the resistance locus *Rfo-Sa1*;
2. BULK SS includes 18 fully susceptible RILs, with a disease resistance ratio of 0 and in which the locus *Rfo-Sa1* is lacking;
3. BULK PR includes 17 partially resistant RILs, in which the locus *Rfo-Sa1* is lacking but with a resistance ratio ranging from 30 to 100%.

### 2.5. BSA-Seq Alignment of Bulked Sequences to Reference ‘67/3’

The two bulks of reads PR (Partially Resistant) and SS (Fully Susceptible) were aligned with Bowtie2 ([53]; tolerance of max 2 mismatches per reads) to the ‘67/3’ eggplant reference Version3 (V3) genome [35], available at http://www.solgenomics.net (accessed on 20 March 2020). Furthermore, forward reads from ‘67/3’ V3 genome and 35X reads from ‘305E40’ were mapped on the reference genome as positive and negative controls, respectively. Default V3 GFF3, plus an additional Augustus annotation not subjected to masking of TE and repeat regions, were loaded on the Integrative Genomic Viewer (IGV) [54] browser, thus allowing integration of gene and TE visualization with the read alignment context in the genomic regions considered. Differential regions of interest were estimated based on a combination of cues, including, in the case of virtually identical reads, the ratio of PR versus SS reads, or, in the case of read heterogeneity (as evidenced by SNP abundance in mapped reads), coherence with the PR reads set. In addition, the QTL confidence intervals on CH11 were analyzed with the SnpEff v4.3 program [55], to infer the potential effect of SNP/Indel identified on candidate genes for the resistance trait. The effect of each polymorphism in ‘305E40’ with respect to the reference ‘67/3’ was classified into four classes: (1) modifier effect, as variants located outside genes (non-transcribed regions or introns); (2) low effect, as synonymous variants in coding regions; (3) moderate effect, as variants altering the aminoacidic sequence; and (4) high effect, as variants changing frameshift, thereby introducing/eliminating stop codons or modifying splice sites. Finally, to identify the best candidate genes, functional annotations pinpointing high-confidence genes with a defense role, as well as evidence from expression levels found in the previously published ‘67/3’ RNA-seq data of 16 tissues, including roots [35], were considered.

### 2.6. De Novo Assembly of ‘305E40’ Line

The 35X Illumina sequencing reads previously submitted to the NCBI Sequence Read Archive are available under accession number SRP078398. Draft de novo assembly (hereafter named “*asm_305*”) of the 35X Illumina sequences was performed using the software Soapdenovo2 ([56], version 2.04) in multi-kmer mode (kmers: 43–91) and an average insert size of 200. The draft assembly resulted in a total of 1,667,559 contigs (N50 value 7937) for a total of 1.155 Gb; of these, 141,312 contigs longer than 1 Kb were subjected to further analysis. The assembly was uploaded at https://figshare.com/account/articles/19778923 (accessed on 10 February 2022).

### 2.7. BSA-Seq Alignment of Bulked Sequences to the Reference Genome Asm_305

The two bulks of the RR (Fully Resistant) and SS (Fully Susceptible) Illumina reads were aligned with Bowtie2 ([53], tolerance of max 2 mismatches per reads) to the *de novo* assembled reference (*asm_305*). Forward reads from ‘67/3’ and 35X ‘305E40’ sequencing were mapped on the *asm_305* reference as negative and positive controls, respectively. 

To prevent artefact-related issues and false-positive covered regions, contigs longer than 1000 bp were further filtered based on the following criteria: a minimum of 50 reads/kb in at least one of RR or SS mapping bulks and a mapping ratio (RR vs. SS) of at least 16. Differentially represented contigs were subjected to gene model identification by implementing Augustus version 3.1 [57], trained with tomato. Identification of both complete and partial CDS was conducted, resulting in 1443 contigs with at least one predicted CDS. As the region putatively containing the QTL *FomCH02* was not physically localizable, the entire sequence of CH02 was considered for the BSA-seq analysis as described above, and in this case, being the ‘305E40’ line responsible for the full resistance to *Fom*, the identification of differentially represented regions was based on a combination of cues relating to RR vs. SS abundance ratio and visual assessment of the amount of read mismatches. Genetic differences between the candidate gene sequences in the ‘305E40’ genotype compared with their orthologues in ‘67/3’ reference genome were identified using the SnpEff v4.3 program [55], to infer the potential effect of SNP/Indel identified on candidate genes for the resistance trait. 

### 2.8. Expression Analysis of Candidate Genes

The expression levels of the best candidate genes identified on chromosome 11 were retrieved from the previously published ‘67/3’ RNA-seq data [30] of 16 tissues, including roots.

The primer sequences to amplify candidate genes on chromosome 02 were retrieved by in silico analysis and CDS sequence predictions within the induced contigs and are detailed in Table 1. For the molecular analysis, the available experimental dataset was the same as already described in Barbierato et al. [39]. RT-qPCR analysis was performed in a 72-Well Rotor with Rotor-Gene RG-6000 (Corbett Research) using GoTaq^®^ RT-qPCR Master Mix by PROMEGA. The reaction containing 1.0 μL of previously diluted cDNA (1:20), from 0.2 μL to 1.0 μL of primers (1 μM each), 5 μL of GoTaq^®^ RT-qPCR Master Mix and Rnase-Free water up to the final volume of 10 μL. All reactions were performed in triplicate with three biological replicates, and no-template samples were included in all the analyses as negative controls. GAPDH was used as a reference gene [58]. Standard curves for each primer pair across a 5-fold dilution series of pooled diluted cDNA amplified in technical triplicate were calculated. Primers, based on the available ‘305E40’ sequences by Primer 3 software (https://bioinfo.ut.ee/primer3-0.4.0/primer3/, accessed on 16 March 2021), were developed (Table 1).

## 3. Results

### 3.1. Phenotypical Score and Statistical Analysis

**Figure 2:**
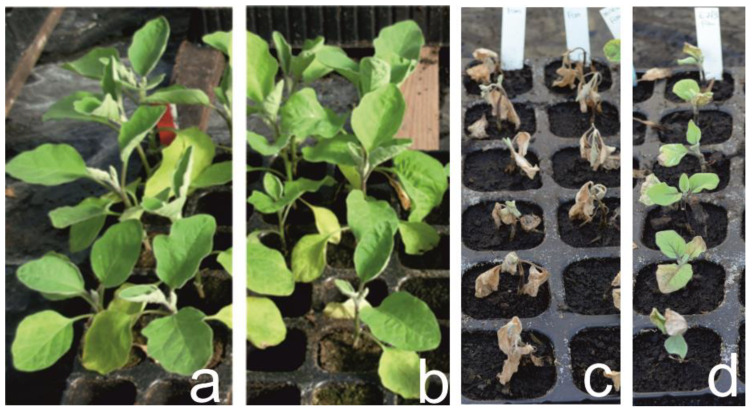
Plantlets of line ‘305E40’ (**a**), of the F_1_ hybrid (**b**), of two full sensitive lines ‘DR2’ and ‘Tal1/1’ (**c**) and of ‘67/3’ (**d**) at 30 DAI after inoculation with *F. oxysporum*.

The line ‘305E40’ and all ‘305E40’x’67/3’ F1 plantlets carrying the locus *Rfo-Sa1* exhibited complete resistance against *Fom* (Figure 2a,b, respectively) as well as the wild donor species *S. aethiopicum*, whose plantlets were completely symptomless at 30 DAI after inoculation (Table 2).

**Figure 3:**
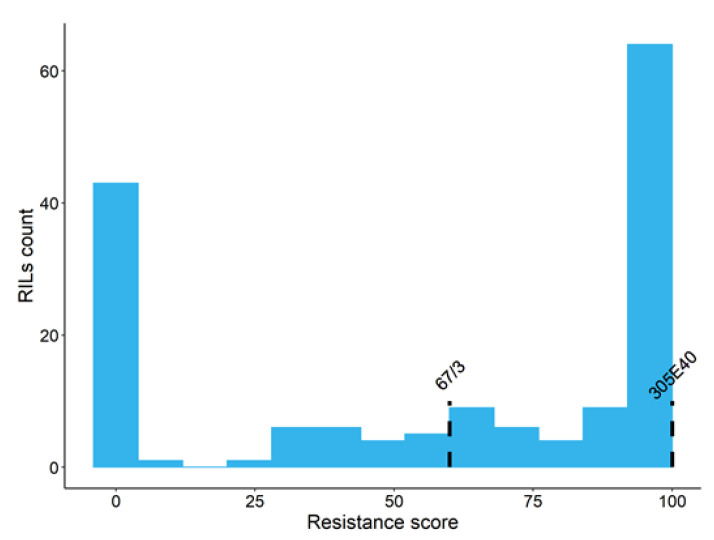
Distribution of the resistance score in the RIL population. The scores values of the two parental lines ‘67/3’ and ‘305E40’ are highlighted with dashed lines.

Conversely, the *Fom*-susceptible control, line ‘Tal1/1’, which is one of the recurrent parents for selecting the ‘305E40’ breeding line after the somatic hybridization [*S. melongena+S. aethiopicum*] confirmed its full susceptibility to the pathogen, so that all inoculated plantlets were completely wilted and died (Figure 2d). All the plantlets belonging to the ‘67/3’ (Figure 2c) parental line survived to *Fom* inoculation, although they showed an average resistance ratio of 60% due to reduced growth and yellowish leaves with respect to the mock inoculated plantlets of the same line (Table 2). Unfortunately, 11 of the 168 total RILs used to build the GBS-based map did not germinate. The distribution of the resistance ratio to *Fom* among the 157 RIL progeny is displayed in Figure 3. Fifty-eight RILs were completely resistant (Score = 100), while 42 were susceptible (score = 0) after *Fom* inoculation. Most of the remaining 57 lines displayed a resistance ratio above 50, with an average disease ratio within the entire RIL population of about 60%. A highly significant genotypic effect was detected for the *Fom* resistance trait (Supplementary material Table S1_sheet 1), also confirmed by a very high heritability value (h = 0.98). Sixty-six transgressive lines were observed only with respect to the most susceptible parent ‘67/3’ (Table 2).

### 3.2. QTL Mapping

The *Fusarium oxysporum* resistance analysis allowed for the identification of two associated regions on chromosomes 2 and 11, named *FomCH02* and *FomCH11*, respectively (Table 3).

The strongest QTL, identified on chromosome 2 and named as *FomCH02*, explained ~47% of PVE and the female parental line ‘305E40’ contributed the allele with the positive effect. The second major QTL (*FomCH11*), located on CH11, explains about 26% of variance (PVE) with a LOD value of 19 and the male parent ‘67/3’ contributed the allele with the positive effect. Finally, 8% of the variance was explained by the interactions between the two QTLs detected.

**Figure 4:**
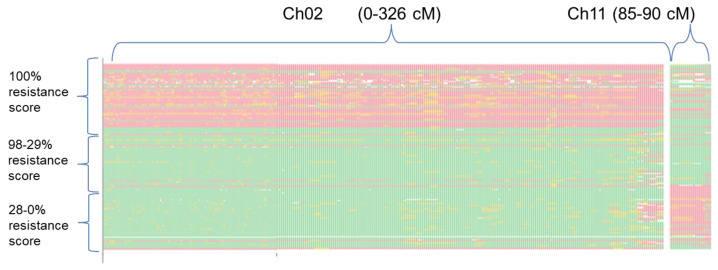
Haplotype distribution in the RIL population of all GBS markers mapped on CH02 (0–326 cM) and CH11 (85–90 cM) was ordered according to their resistance scores (on the left panel, descending from 100% to 0). Each line represents RIL progeny. In red, haplotype of ‘305E40’; in green haplotype of ‘67/3’; in yellow heterozygous markers; in white, missing data.

To confirm the involvement of *FomCH02* and *FomCH11* in the *Fom* resistance trait, the haplotype distribution of all the GBS markers belonging to the genomic regions underlying the two QTLs was assessed in all the RILs after manual ordering in accordance with their resistance scores. Regarding *FomCH11*, a region spanning between 85 and 90 cM on chromosome 11 and corresponding to a physical interval of 5Mbp (from 64.5 to 69.5 Mb) was considered. The physical extension of *FomCH02* was difficult to determine due to the highly discordant ordering of the molecular markers according to their physical mapping along the V3 sequence of 67/3 compared to their position in the genetic map. Therefore, we decided to include all the GBS markers belonging to the entire CH02 group in this investigation. This choice allowed us to highlight a highly conserved region of CH02 spanning from 0 to 310 cM out of the total 326 cM, where the haplotype resulted continue and identical to that of ‘305E40’ (Figure 4) in forty-seven out of 58 (47/58) completely resistant RILs, plus 2/58 RILs in which the same region was fully heterozygous. Five full-resistant RILs exhibited a slightly fragmented haplotype in CH02, which deserves better characterization. Lastly, 4/58 RILs harbored a ‘67/3’ haplotype on CH02, but, concurrently, showed the haplotype of ‘67/3’ within the genomic region underlying *FomCH11*; therefore, their resistance score could likely be due to a transgressive effect of this QTL, mimicking the “full resistant” phenotype. Among the RILs with R scores less than 100%, four still displayed a slightly fragmented ‘305E40’ haplotype in CH02, which would deserve a better characterization. All the remaining RILs with a score ranging between 98% and 28% exhibited a ‘67/3’ haplotype both in CH02 and in the region underlying the *FomCH11*, therefore inherited the partial resistance trait harbored from the ‘67/3’ line. All the RILs with resistance scores below 28% showed a ‘67/3’ haplotype in CH02 and a ‘304E40’ haplotype within *FomCH11*, therefore seeming to have inherited neither of the favorable trait haplotypes from both parents.

### 3.3. BSA-Seq Analysis and Candidate Gene Identification in the FomCH11 Region

**Figure 5:**
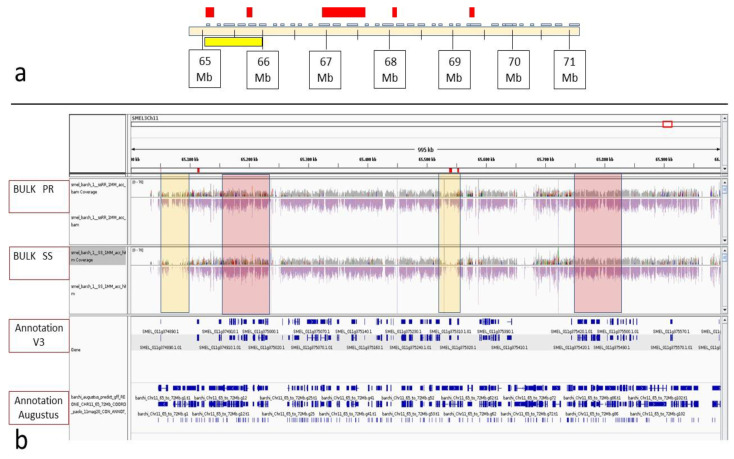
Example of IGV visual score in the region spanning 65 and 66 MB on chromosome 11. (**a**) In the upper panel, the region ranging from 63–71 Mb comprising within the CI of the *FomCH11* QTL is represented. The yellow box depicts the region zoomed in the box below. The red boxes indicate the position of the 5 differentially enriched regions highlighted from BSA-seq analyses. (**b**) Read alignments of the two bulks PR (on the top) and SS (bottom) against the reference sequence of ‘67/3’ are shown, together with the annotated genes according to V3 genome sequence ([35], **top**) and the additional Augustus annotation (**bottom**), also including TE-related genes. The two yellow rectangles highlight two differentially enriched regions between bulks of PR and SS (i.e., with different read coverages detectable when comparing upper and lower panels), while the pink rectangles highlight the position of two non-enriched ones.

The genomic region within the *FomCH11* confidence interval was subjected to a finer characterization and revealed minor inversions and other embedded structures still present in the V3 genome [35], evidenced by slight discrepancies between the GBS marker order and their physical location along the chromosome. Regarding the BSA-seq analysis, two bulks of reads from selected PR and SS RILs were created and aligned, together with reads from ‘67/3’ and ‘305E40’ as positive and negative controls, respectively, to the ‘67/3’ eggplant V3 reference genome focusing on the *FomCH11* region spanning 5 Mb (from 64.5 to 69.5 Mb), which contains 278 annotated genes (Figure 5a). The differentially represented genomic regions were estimated based on a combination of cues, including, in most cases, a differential (higher) ratio of mapping PR versus SS reads (Figure 5b) within a predicted region as CDS, but also the presence of differential mismatches of PR vs. SS reads in a region covered by a comparable amount of mapping reads between the two bulks. Indeed, such poor coherence between the reads would be consistent with SS reads arising from close paralogous gene mapping artefactually due solely to mismatch tolerance. 

In addition, the presence of a CDS was among the cues considered to select the most interesting regions. IGV visual inspection led to the identification of five differentially enriched genomic regions underlying the QTL, characterized by differential coverage in reads from RR compared to the SS bulk (Table 4). In these regions, nine candidates, annotated as resistance genes, were highlighted. To infer functional annotation and the potential deleterious effect of the candidate genes responsible for the *Fom*-resistance trait in ‘305E40’ compared to the reference ‘67/3’, all the annotated genes within the *FomCH11* confidence interval were further analyzed using SnpEff v4.3 program (Supplementary material Table S2). All nine candidate genes highlighted within the five differentially represented regions exhibited an SNP effect from “moderate” to “high” in the ‘305E40’ variants (Table 5).

Moreover, two additional genes, SMEL_011g374910 and SMEL_011g374920, annotated as ‘Similar to RPP13: Disease resistance protein RPP13 from *A. thaliana*’ and proximal to the differential region “A” (therefore named as “A+” -from 65.150 Mb to 65.168 Mb) were included to the list of best candidates according both to their putative function and the high SnpEff score. In Table 5, the expression levels of the 11 candidate identified genes in sixteen ‘67/3’ tissues, according to previously published RNA-seq data [35], are reported.

### 3.4. De Novo Assembly of ‘305E40’, BSAseq and Candidate Gene Identification in the QTL FomCH02 Region

To better resolve the CH02 region introgressed from the allied species *S. aethiopicum* in ‘305E40’ containing the *FomCH02* QTL, a new genome assembly of the available 35X Illumina reads for this line was performed, leading to the generation of a de novo assembly more suitable as reference (hereafter named *asm_305*). It covers 1.155 Gb, including 1,667,559 contigs (CG), with an N50 of 7937 bp and an N90 of 159 bp. More in detail, in the *asm_305* assembly, 263.683 CGs have a length > 300 bp, of which 202.945 > 500 bp, and 141.312 > 1000 bp. 

**Figure 6:**
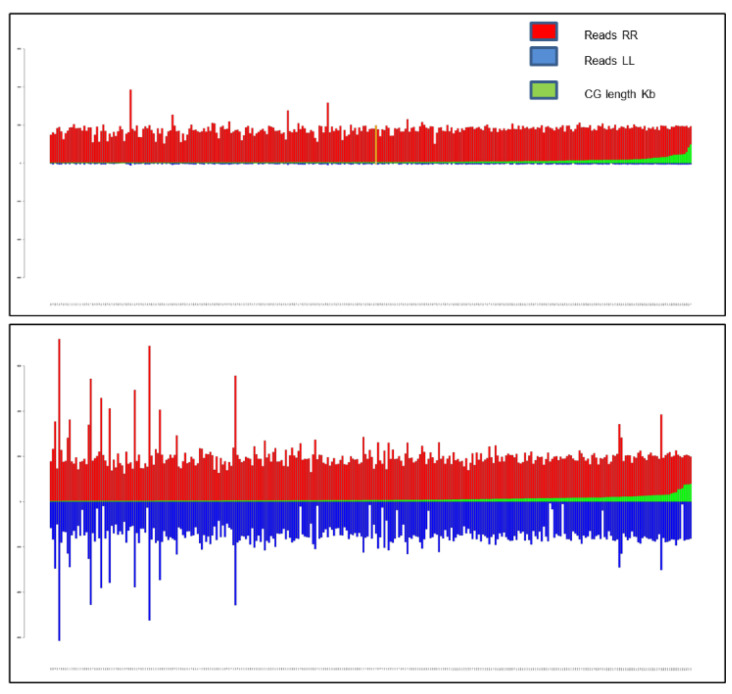
Graphical representation of selected contig coverage in the *asm_305* assembly. Upper panel: Contigs exhibiting a ratio of mapped RR vs. SS reads of a least 30 (*log2* > 4.90) are shown. Lower panel: contigs exhibiting a ratio of RR vs. SS reads < 30 are shown. For both panels, contigs are ordered by increasing the length along the X axis. RR, SS and contig length are represented as red, blue and green bars, respectively.

To identify scaffolds and contigs highlighting differentially covered regions in which genes responsible for the full resistance trait to *Fom* might be included, the *asm_305* was employed as a reference for the BSAseq mapping of bulked reads from 18 SS and 28 RR RILs. Among the contigs with a length > 1000 bp and a tolerance of >50 reads/kb in at least one RR or SS mapping bulk, a subset of 1838 “enriched” contigs/scaffolds with *log2* > 4 RR vs. SS was identified (total scaffold length of 9.36 Mb). Considering a more stringent tolerance, 691 contigs (7.43 Mb) with a length >3 Kb with *log2* > 4, of which 306 (3.01 Mb) with *log2* > 4.90 (equivalent to a 30-fold enrichment in RR vs. SS reads), have been also identified (Figure 6). Among this last dataset, the scaffold #67320 can be highlighted, which contains the sequence amplified by the *Rfo-Sa1* markers. 

The *log2* > 4 over-represented subgroup of 1838 contigs > 1 Kb was employed for the in silico analysis and CDS prediction. BLASTn comparison between all the identified CDS in the enriched contigs versus *S. aethiopicum* assembled transcripts [59] led to the identification of nine candidate genes (RES2-RES10; Table 6) with a complete or nearly complete match with *S. aethiopicum* and a tolerance of 1e^−100^. A BLASTn search of the contigs/scaffolds belonging to the *asm_305* containing these genes led to the identification within the *S. aethiopicum* draft genome [60] of corresponding scaffolds with complete or nearly complete matches (Table 6). SNP Eff analyses were not successful in a great part of candidates, as the orthologous sequence was not found in ‘67/3’, but, when it was possible, the analysis revealed polymorphisms with severe effect between ‘67/3’ and ‘305E40’ sequences. Furthermore, a search for orthologues in the eggplant V3 genome of the twenty-five tomato candidate genes, identified through synteny analysis by Miyatake et al. [20] within the confidence interval of FomCH02, yielded four annotated genes. One of these, showing 99% homology with an *S. aethiopicum* transcript, was added to the list of our candidate genes (indicated as RES1 in Table 6). A comprehensive list of the 10 best candidate genes is reported in Table 6.

**Table 6:** List of best candidate genes identified among the overrepresented bulks of reads from resistant RILs and with the best match with transcriptome data of *S. aethiopicum*. For each gene, the *305_asm* scaffold or contig, the matching *S. aethiopicum* scaffold (from Gramazio et al. [59]), the percentage of homology and a putative gene prediction are indicated.

Candidate Genes	Query	Scaffold on *S. aethiopicum transcriptome*	Identities	Predicted Function Based on Domains Analysis Performed via NCBI Platform	Scaffold on *S. aethiopicum* Pangenome
*RES1*	SMEL_002g157480.1 (Miyatake et al. [20], orthologous of SOLYC02G032200.2	SAUC48279_TC01 Length = 3745	3392/3418 (99%) Strand = +/+	Encoding a putative TMV resistance protein N-like LOC102604931, transcript variant X2	scaffold3814_cov65 (978531-977633) lenght:898 strand (+/−)
*RES2*	C7021905__121_4977 (348 letters)	SAUC67459_TC01 Length = 578	348/348 (100%) Strand = +/−	chaperonin	scafold150403_cov62 (904702-904125) lenght:577 strand (+/−)
*RES3*	C7104747__218_9061 (1767 letters)	SAUC05724_TC02 Length = 1311	915/915 (100%) Strand = +/+	cysteine-rich RLK (RECEPTOR-like protein kinase) 8	scafold150406_cov62 (122904-121605) lenght:1299 strand (+/−)
*RES4*	C7123897__247_10432 (1176 letters)	SAUC85719_TC01 Length = 1281	194/194(100%) + 807/807(100%) Strand: +/+	Putative late blight resistance protein homolog R1A-3	scaffold149207_cov61 (473206-471925) lenght:1281 strand (+/−)
*RES5*	scaffold131120__543_23320 (630 letters)	SAUC54187_TC01 Length = 581	312/312 (100%) Strand: +/+		scaffold4400_cov64 (319724-320299) lenght:575 strand (+/+)
*RES6*	scaffold151247__813_35520 (1935 letters)	SAUC60998_TC03 Length = 2636	986/986(100%) + 719/721(99%) Strand = +/+		scaffold149470_cov62 (278029-278886) lenght:857 strand (+/+)
*RES7*	scaffold161031_1035_45870 (1362 letters)	SAUC62185_TC01 Length = 517	517/517 (100%) Strand = +/+	Protein transparent testa 12-like	scaffold149494_cov61 (246517-247031) lenght:514 strand (+/+)
*RES8*	scaffold4270_1160_51181 (3702 letters)	SAUC68094_TC01 Length = 4264	2697/2697(100%) + 974/974 (100%) Strand = +/−	Putative late blight resistance protein homolog R1B-14-like [Solanum lycopersicum]	scaffold872_cov63 (353791-349798) lenght:3993 strand (+/−)
*RES9*	scaffold160330_1010_44805 (327 letters)	SAUC18225_TC01 Length = 741	327/327(100%) Strand = +/−		scaffold149207_cov61 (183171-183700) lenght:529 strand (+/+)
*RES10*	scaffold83272__1378_60779 (375 letters)	SAUC44781_TC01 Length = 543	364/364(100%) Strand = +/−		scaffold150551_cov61 (32877-32417) lenght:460 strand (+/−)

### 3.5. Expression Analysis of Candidate Genes for FomCH02

**Figure 7:**
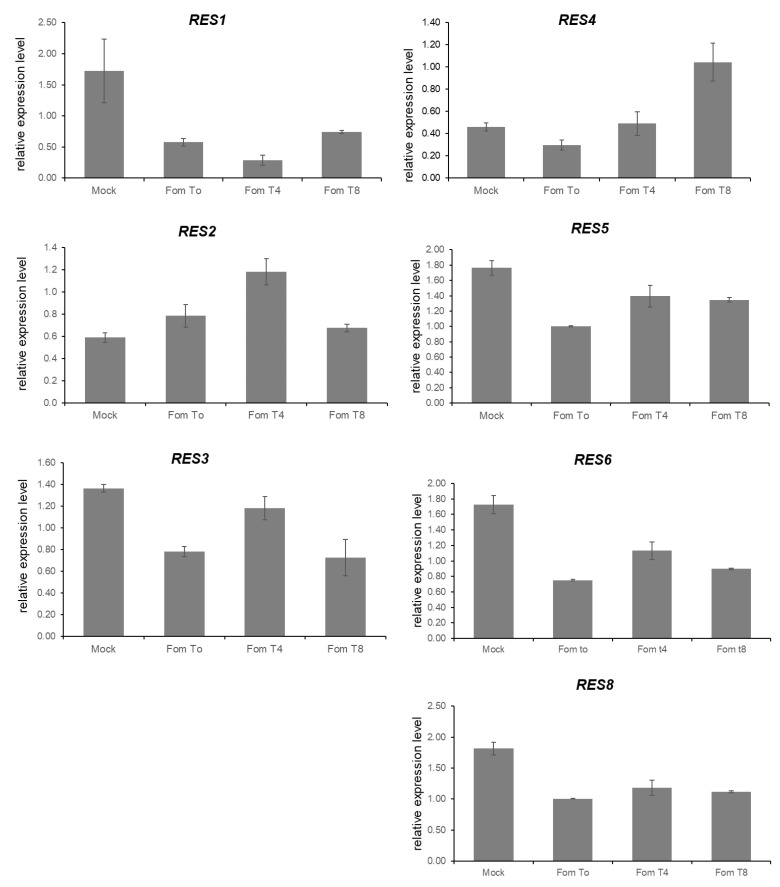
RT-qPCR analysis of *S. melongena* candidate transcripts using RT-qPCR. Relative quantification of 7 candidate RES genes. Values are expressed relative to the GAPDH used as a reference gene and are the averages of three biological replicates (*n* = 3). Mock control indicates not inoculated samples, *Fom* t0, t4, t8 indicates inoculated samples at 0-, 4- and 8-h post infection.

Primers for qRT-PCR were developed on the consensus-predicted CDS sequence (Table 1) of the 10 genes (RES1-RES10) with the highest match with the *S. aethiopicum* transcript. For 7 out of 10 candidate genes (RES1, RES2, RES3, RES4, RES5, RES6 and RES8), preliminary expression data through real-time qPCR analysis was successfully obtained in ‘305E40’ cDNA samples of roots at different timepoints after inoculation with *Fom*, while about the other three genes (RES7, RES9, RES10) RT-qPCR always led to multiple peaks in melting or was not reliable at all. These 7 genes (RES1, RES2, RES3, RES4, RES5, RES6 and RES8) are all expressed at T0 both in *Fom*- and mock-inoculated roots (Figure 7) and their expression at different time points in response to fungal inoculation is described in Figure 7.

## 4. Discussion

*Fusarium oxysporum* f. sp. *melongenae* (*Fom*) is one of the most damaging and widespread pathogens of eggplant, due to its soil-borne nature and persistence for several years in the field. Crop rotation, fumigation and fungicide applications can reduce the risk of infection. However, such countermeasures are not highly effective solutions and are also environmentally impactful [61,62]. Some more eco-friendly alternatives to the chemical treatments could be represented using antagonistic rhizospheric fungi, which proved effective to some extent in contrasting the effect of *Fusarium* in eggplant [63,64]. Among the best available alternatives, however, is the development of *Fom*-resistant cultivars by exploiting the natural diversity of the cultivated eggplant, including the sources of resistance existing within its related species [19]. The newly developed resistant cultivars could determine a more environmentally friendly and efficient reduction of pathogen growth, minimal damage to the host plant, and zero input of pesticides from the farmers.

However, a breeding program for resistance involving the introgression of resistance genes from wild/allied species into the eggplant genome by repeated backcrossing is a long-term process that includes many cycles of backcrosses. Therefore, a deep understanding of plant–pathogen interaction, together with the characterization, functional study, cloning and genetic transformation or editing of resistance genes, could help researchers speed the selection for the development of resistant varieties [65].

### 4.1. QTL Mapping

In this paper, to spot and characterize the genomic regions underlying the *Fusarium oxysporum* resistance traits, as well as to identify candidate genes involved in this trait, we assayed the plant responses to *Fom*-inoculation of an eggplant RIL population derived from the cross between ‘305E40’x’67/3’ recently used for developing a high-quality GBS-based map [40]. Both parents of the RIL population are known to carry a resistance trait to *Fom:* the breeding line ‘305E40’carries the locus *Rfo-sa1* introgressed from *S. aethiopicum*, which confers complete resistance to the soil-borne fungus [30], while the breeding line ‘67/3’ carries a source of partial resistance trait that was unexpected in the cultivated eggplant germplasm [18]. The exploitation of these traits would be of extreme interest for developing new resistant breeding lines carrying both *Fom* resistance traits, considering that pyramiding of multiple resistance genes in the same varieties would strengthen the host response and should provide a broad spectrum and more durable resistance to *Fom* [66,67]. 

All the ‘305E40’x’67/3’ F1 plantlets revealed full resistance to *Fom*, further confirming the dominant behavior of the full resistance trait introgressed from the wild species and associated with the *Rfo-Sa1* locus with respect to the partial resistance one triggered by ‘67/3’. Data of replicated inoculations of each RIL were successfully employed to identify two major QTLs on chromosomes CH02 (*FomCH02*) and CH11 (*FomCH11*) associated with complete and partial resistance trait to *Fom*, respectively, which confirmed, better defined and also narrowed the regions underlying the two major QTLs already spotted in the same position using the F_2_ population from the same cross and the RAD-Tag-based map [18]. Despite the availability of the newly developed ultra-dense genetic map and of a sequenced reference genome of ‘67/3’ [35], it was still difficult to determine the true physical extension of the confidence interval of the *FomCH02 QTL*, as in the entire chromosome 02 the order of GBS-based molecular markers according to the map is still extremely discordant with their physical position mapped in the V3 sequence of 67/3. The presence of the introgressed wild fragment in line ‘305E40’ probably hampered a correct pairing of chromosomes during meiosis, resulting in a distorted segregation ratio of markers and, as consequence, to a wrong marker position assignment along the map or an inaccurate chromosome scaffolding.

The major QTL *FomCH11*, is co-localizing with the major QTL associated with the partial resistance trait already spotted (despite with broader confidence interval) by Barchi et al. [18].

### 4.2. BSA-Seq Approach

The development of next-generation sequencing (NGS) and reliable bioinformatics tools boosted segregant population analyses (including BSA), expression profiling, and the construction of polymorphism databases to assist QTL identification [68]. The Bulked-Segregant Analysis coupled with Whole Genome Sequencing (BSA-Seq) technique and linkage mapping approaches was successfully exploited in many crops for narrowing the CI of QTLs, developing molecular markers and assisting identification and map-based cloning of candidate genes linked to several traits of interest [69,70,71,72,73]. An even more localized or targeted approach can be set up if QTLs and genomic regions controlling a trait of interest are already available. Therefore, a high-quality assembled genome serving as the reference sequence in genotype calling is an essential step in BSA-Seq data analysis. For most species, however, such high-quality assembled sequences are available only for a single or a limited number of breeding lines in many plant species. Therefore, the parent included in a BSA-Seq experiment that lacks a high-quality assembled genome might be sequenced via NGS to determine the allelic variants compared to a reference genome.

Here, to deeply investigate the genomic regions underlying two *Fom*-resistance QTLs in eggplant, we took advantage of the availability of a high-quality ‘67/3’ annotated genome sequence, as well as a 35X resequencing of the other parent ‘305E40’ and the RILs to apply a targeted BSA-seq approach. The two *Fom*-resistance QTLs are carried by different parents, and as one of them seems localized in a unique genomic region of ‘305E40’ and absent into the ‘67/3’ reference genome, two independent rounds of BSA-seq were performed. Bulked reads from different RIL subsets, grouped according to their resistance score, were aligned for each QTL to the appropriate reference genome, enabling us to highlight differential enriched genome regions useful for identifying the most reliable candidate genes responsible for both sources of *Fom*-resistances. This strategy allowed us to spot differential genomic regions between the parents putatively involved in controlling either partial or complete resistances on CH11 and CH02, respectively.

### 4.3. Differentially Enriched Regions and Candidate Gene Identification within FomCH11 QTL

The alignment to the ‘67/3’ (itself a parent of the cross-employed) reference genome of bulked sequence reads from partially resistant (PR) and fully susceptible (SS) RILs allowed the identification of five differentially enriched regions containing eleven putative candidate genes within the confidence interval of the *FomCH11* QTL. Among them, three candidates, including a putative late-blight resistance protein and two homologs of RPP13, an NB-ARC domain-containing disease resistance protein conferring resistance to *Peronospora parasitica* in *A. thaliana* [74,75], were selected according to SNPeff output and expression data for future functional characterization through gene silencing and/or overexpression. Furthermore, following the comparison of PR and SS reads in the regions carrying the candidate genes, a set of molecular markers for finer mapping of the QTL *CH11Fom* locus and for MAS purposes may be developed.

### 4.4. De Novo Assembly of ‘305E40’, BSAseq and Candidate Gene Identification within FomCH02 QTL

Regarding the *FomCH02* QTL, a BSA-seq characterization similar to that performed for *FomCH11* using the ‘67/3’ V3 genome as a reference was not conceivable due to the *S. aethiopicum* origin of the genomic region underlying this QTL, which is exclusive of the ‘305E40’ parent and entirely lacking in ‘67/3’ [32]. The parental line possessing the favorable allele, ‘305E40’, is a stable double-haploid line derived from the androgenetic progeny of a somatic hybrid consisting of the fusion between protoplasts of eggplant and *S. aethiopicum* [26] bearing a segment derived from the allied parent located in the distal portion of chromosome 02 [36], which includes the locus *Rfo-sa1* associated with the resistance trait [30]. A previous study already evidenced that in the tetraploid somatic hybrid *S. melongena* + *S. aethiopicum* gr *gilo*, from which the ‘305E40’ line originated, both tetrasomic and disomic inheritance of ISSR and isoenzyme markers occurred [76]. Likely, the non- or poorly-recombinant *S. aethiopicum* introgressed fragment in CH02 was stably fixed in the ‘305E40’ because of both the selection for the resistance to *Fom* during the backcrossing cycles and the final step of another culture. As also confirmed in this paper, when evaluating the haplotype of all fully resistant RILs, this genomic region did not or very hardly show genetic recombination [77], making mapping and cloning extremely difficult, probably due to gene megaclusters flanked by other inactive copies. 

Thus, the short Illumina read sequences already available for ‘305E40’ [30] were *de novo* assembled to define a *305_asm* reference genome of the line ‘305E40’ and then coupled with a targeted BSA-Seq technique. This strategy allowed us to develop a more appropriate reference genome to which the RIL sequences, pooled according to their disease resistance scores, were aligned. By comparing reads from resistant (RR) and susceptible (SS) RIL bulks, exclusive chromosomal regions from ‘305E40’ absent in the ‘67/3’ reference genome were revealed, resulting in differentially covered by the two bulks of reads. 

Deep investigation of these exclusive genomic regions enabled the identification of 1838 contigs/scaffolds containing differentially enriched regions, which were crucial in the search for unique candidate genes associated with the resistance trait introgressed from the wild species.

The comparison between all the identified CDS in the enriched contigs versus the *S. aethiopicum* assembled transcripts [59] and draft genome [60] led to the identification of nine candidate genes displaying a complete or nearly complete match with the allied species from which the line ‘305E40’ was developed. The candidate genes, when possible, were annotated as resistance genes. A tenth candidate gene (*RES1*) was added to this list after orthologous search between the eggplant V3 genome, the *305_asm* assembly and the tomato candidate genes suggested by Miyatake et al. [20]. *RES1*, annotated in eggplant as *SMEL_002g157480.1* and encoding for a putative TMV resistance protein, displayed 99% homology with an *S. aethiopicum* transcript. A deeper comparison between *RES1* allelic variants in ‘67/3’ and ‘305E40’ lines is ongoing to identify and validate the effect of nucleotide polymorphisms on gene function.

The qRT-PCR expression analysis of the candidate genes from the ‘305E40’ assembly by confirmed that they are not annotation artefacts.

Complementary DNA amplification from ‘305E40’ root yielded in seven genes the predicted fragments, whose amplification was not obtained by the cDNA from the *Fom*-sensitive line (data not shown). qRT-PCR revealed that all these genes are expressed at T0 in both *Fom-* and mock-inoculated roots, as observed for many R-genes [78]. Afterwards, at different time samplings, their expression frequently slightly increased 4 h after the *Fom*-inoculation. Among them, a candidate deserving a further functional characterization is *RES8*, identified by combining the results obtained from gene annotation within the differentially enriched regions in the *asm_305* de novo assembled genome together with the comparison to the *S. aethiopicum* transcriptome [59] and draft genome [60] and our gene expression analysis. This gene, belonging to the *305_asm* Scaffold 4270, encodes for a putative late blight resistance protein; a preliminary phylogenetic study (data not shown) revealed its high homology to late blight resistance genes from tomato and *S. demissum* [79]. The *RES8* sequence is complete in our annotation and displays a perfect match with that of *S. aethiopicum*. Blast comparison of this sequence to the ‘67/3’ genome did not reveal orthologous genes, leading to the hypothesis that *RES8* comes from the allied species and can be eligible as the best candidate.

## 5. Conclusions

The genes candidate as playing a key role in the plant response to *Fom* infection reported here may be exploited to develop molecular markers to efficiently and quickly transfer the involved QTLs into elite eggplant germplasm. However, a finer identification of the genomic region involved in the *Fom* resistance from *S. aethiopicum* would require an effective long read sequencing strategy of the ‘305E40’ line, along with an additional enrichment stage of the genomic regions with repeated sequences and resistance genes in tandem, as well as a targeted novel RNA-seq experiment. Moreover, the genomic characterization of RILs revealed that five of the *Fom*-resistant (RR) harbor reduced portions of the QTL on CH02, which might be re-sequenced with the aim of narrowing down the genomic region, easing the cloning of resistance gene(s). Likewise, the RILs mimicking a fully resistant phenotype despite not harboring the *FomCH02* QTL, but the ‘67/3’ haplotype in the *FomCH11* QTL could represent the lines carrying the most effective partial resistance trait worthwhile to be re-sequenced for identification and isolation of the underlying genes. Finally, the best performing RILs for full and/or partial resistance traits will be employed for breeding purposes to introgress and pyramid the two *Fusarium* resistance QTLs into élite eggplant germplasm aiming to accomplish a more durable resistance. 

## Figures and Tables

**Table 1:** Primers developed for RT-qPCR analysis (detailed information is reported in Table 6).

Gene Abbreviation	Oligo Sequences for RT-qPCR
RES_1	RES_1_FW 5′_TGGCAGAATCTCCACAACCT_3′ RES_1_RV 5′_GATGATGAAGGACTGCTCGC_3′
RES_2	RES_2_FW 5′_ACCAGCACTGATCTGTCTCC_3′ RES_2_RV 5′_TATGACCGGTCCCTTTTCCC_3′
RES_3	RES_3_FW 5′_AGTACAAGGGAAGCCGTGAG_3′ RES_3_RV 5′_GAGCAGCATCAGATCAGCAC_3′
RES_4	RES_4_FW 5’_ACGGAACTAGAGCGACAACA_3′ RES_4_RV 5’_TAGCCTTGCCTCTATCCTGC_3′
RES_5	RES_5_FW 5′_CCGCCAATTCACTGCGTAG_3′ RES_5_RV 5′_TTGTATCCTCCTCCTCGCTG_3′
RES_6	RES_6_FW 5′_TTTGAGCTGTTGGGCCAATC_3′ RES_6_RV 5′_CCGTGGTGCATTATAGCCAC_3′
RES_7	RES_7_FW 5′_AATGGGAAGTGCAGTGGAGA_3′ RES_7_RV 5′_GGGGAAGTTGGCAGCATAAG_3′
RES_8	RES_8_FW 5′_ACCAGGTTAAGTTACAGCTCTGA_3′ RES_8_RV 5′_ACCCCTTTCCAGACACATCA_3′

**Table 2:** Disease score of the parental lines, of the two control lines for complete susceptibility (‘Tal1/1’) and resistance (*S. aethiopicum*), the F_1_ hybrid (‘67/3’x’305E40′) and the mean value in the RIL populations. Skewness, kurtosis, broad sense heritability and transgressive genotypes for the trait in study with respect to both parental lines are also reported.

Disease Score (Mean ± SD)						Skewness	SE	Kurtosis	SE	Heritability	Transgr. vs. ‘305E40’	Transgr. vs. ‘67/3’
‘305E40’	‘67/3’	*S. aeth*	‘Tal1/1’	F_1_	RIL Population							
100 ± 0	60 ± 0	100 ± 0	0 ± 0	100 ± 0	59.8 ± 42.0	−0.455	0.193	−1.502	0.384	0.98		66

**Table 3:** QTL identified as associated with *Fusarium* resistance. Chromosomes (Chr), peak marker position (cM), LOD scores, percentages of phenotypic variance explained (PVE%), estimated additive effects (Add. Eff.), and the confidence intervals (CI) were also provided.

QTL Name	Chr	cM	LOD	PVE (%)	Add. Eff.	CI (cM)	
						Start	End
*FomCH02*	2	222.7	29.03	46.47	−30.9	221.66	223.74
*FomCH11*	11	88.5	18.98	25.72	14.61	86.61	90.39
Interaction 2*11			7.42	8.37	13.15		

**Table 4:** List of differentially enriched regions within the confidence interval of QTL *FomCH11*. For each region, extension, number of annotated genes, number of genes annotated as resistance genes and details for each best candidate gene according to the annotation in eggplant genome V3 [35] are reported.

Diff. Enriched Region	Physical Interval in V3	Number of Annotated Genes	Number of Resistance Genes	Gene ID According to Gene Annotation by BARCHI et al. [35]
**A**	65,030–65,120 Mb	2	2	**SMEL_011g374890.1** Similar to At1g58602: Probable disease resistance protein At1g58602 (*A. thaliana*)
				**SMEL_011g374900.1** Similar to RPP13: Disease resistance protein RPP13 (*A. thaliana*)
**B**	65,532–65,553 Mb	2	**2**	**SMEL_011g375310.1** Similar to RPP13: Disease resistance protein RPP13 (*A. thaliana*)
				**SMEL_011g375320. 1** Similar to RPP13: Disease resistance protein RPP13 (*A. thaliana*)
**C**	66,920–67,557 Mb	31	0	
**D**	68,093–68,171 Mb	7	3	**SMEL_011g376860.1** Similar to XA21: Receptor kinase-like protein Xa21 (*O. Sativa* subsp. Indica)
				**SMEL_011g376900.1** Similar to FLS2: LRR receptor-like serine/threonine-protein kinase FLS2 (*A. thaliana*)
				**SMEL_011g376910.1** Similar to FLS2: LRR receptor-like serine/threonine-protein kinase FLS2 (*A. thaliana*)
**E**	69,366–69,410 Mb	7	2	**SMEL_011g377340.1** Similar to R1A: Late blight resistance protein R1-A (*S. demissum*)
				**SMEL_011g377380.1** Similar to R1C-3: Putative late blight resistance protein homolog R1C-3 (*S. demissum*)

**Table 5:** List of the most reliable candidate genes selected within the differentially enriched regions according to SNPeff output, which suggests the severity of the effect potentially provoked by the allelic variant in the ‘305E40’ line with respect to the ‘67/3’ reference gene form. For each candidate gene, the differentially represented regions of provenance are indicated, together with the SNPeff and the expression levels in different tissues of ‘67/3’ including roots retrieved from RNAseq data already available [35].

		Expression Level						
Diff. Region	Gene ID	SnpEff	Roots	Expanded Leaves	Open Flowers	Fruits 2–4 cm	Fruit Stage B	Fruit Stage C
**A**	**SMEL_011g374890.1**	Mod	0	0	0	0	0	0
**A**	**SMEL_011g374900.1**	High	0.716154	12.2523	0	8.27647	3.05559	2.95479
**A+**	**SMEL_011g374910.1**	High	0	5.38543	0	4.35919	4.00833	16.8497
**A+**	**SMEL_011g374920.1**	High	26.9772	25.087	31.93	9.58168	16.8583	50.3871
**B**	**SMEL_011g375310.1**	High	0	0	0	0	0.221595	0.474865
**B**	**SMEL_011g375320.1**	Mod	0	0	0	0	0.636017	0
**D**	**SMEL_011g376860.1**	High	0	0	0	0	0	0
**D**	**SMEL_011g376900.1**	High	0	0	0	0	0	0
**D**	**SMEL_011g376910.1**	High	0	0	0	0	0	0
**E**	**SMEL_011g377340.1**	High	1.31895	0	0	0.761885	1.12618	0
**E**	**SMEL_011g377380.1**	ND	0	0	0	0	0	0

## Data Availability

Raw read data of ‘305E40’ and 5x Illumina sequences for each RIL [30] were submitted to the NCBI Sequence Read Archive and are available under the accession number SRP078398. Further information, including the ‘67/3’ genome assembly, pseudomolecules, annotations, and tracks for the genome browser are available, in downloadable form, on the Solanaceae Genome Network. The assembly was uploaded at https://figshare.com/account/articles/19778923 (accessed on 10 February 2022). The subset of over-represented sequences within de novo sequencing is available from the corresponding authors on reasonable request.

## Supplementary Materials

The following supporting information can be downloaded at: https://www.mdpi.com/article/10.3390/cells11162548/s1. Table S1: Results of ANOVA analyses; Table S2: Lists of all genes with the SNP effect detected in the region the chromosome 11 underlying the *Fomch11 QTL*.
